# Functional interaction between Lypd6 and nicotinic acetylcholine receptors

**DOI:** 10.1111/jnc.13718

**Published:** 2016-08-15

**Authors:** Maria Arvaniti, Majbrit M. Jensen, Neeraj Soni, Hong Wang, Anders B. Klein, Nathalie Thiriet, Lars H. Pinborg, Pretal P. Muldoon, Jacob Wienecke, M. Imad Damaj, Kristi A. Kohlmeier, Marjorie C. Gondré‐Lewis, Jens D. Mikkelsen, Morten S. Thomsen

**Affiliations:** ^1^ Department of Drug Design & PharmacologyUniversity of CopenhagenCopenhagenDenmark; ^2^ Neurobiology Research UnitUniversity Hospital CopenhagenRigshospitaletCopenhagenDenmark; ^3^ Laboratory for NeurodevelopmentDepartment of AnatomyHoward University College of MedicineWashingtonDistrict of ColumbiaUSA; ^4^ Laboratory of Experimental and Clinical NeurosciencesUniversity of PoitiersPoitiersFrance; ^5^ Epilepsy ClinicUniversity Hospital CopenhagenRigshospitaletCopenhagenDenmark; ^6^ Department of Pharmacology and ToxicologyMedical College of VirginiaVirginia Commonwealth UniversityRichmondVirginiaUSA; ^7^ Department of NutritionExercise and Sport & Department of Neuroscience and PharmacologyUniversity of CopenhagenCopenhagenDenmark

**Keywords:** affinity purification, Ly‐6, LY6/PLAUR domain‐containing 6, Lynx, nicotine

## Abstract

Nicotinic acetylcholine receptors (nAChRs) affect multiple physiological functions in the brain and their functions are modulated by regulatory proteins of the Lynx family. Here, we report for the first time a direct interaction of the Lynx protein LY6/PLAUR domain‐containing 6 (Lypd6) with nAChRs in human brain extracts, identifying Lypd6 as a novel regulator of nAChR function. Using protein cross‐linking and affinity purification from human temporal cortical extracts, we demonstrate that Lypd6 is a synaptically enriched membrane‐bound protein that binds to multiple nAChR subtypes in the human brain. Additionally, soluble recombinant Lypd6 protein attenuates nicotine‐induced hippocampal inward currents in rat brain slices and decreases nicotine‐induced extracellular signal‐regulated kinase phosphorylation in PC12 cells, suggesting that binding of Lypd6 is sufficient to inhibit nAChR‐mediated intracellular signaling. We further show that perinatal nicotine exposure in rats (4 mg/kg/day through minipumps to dams from embryonic day 7 to post‐natal day 21) significantly increases Lypd6 protein levels in the hippocampus in adulthood, which did not occur after exposure to nicotine in adulthood only. Our findings suggest that Lypd6 is a versatile inhibitor of cholinergic signaling in the brain, and that Lypd6 is dysregulated by nicotine exposure during early development.

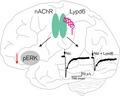

Regulatory proteins of the Lynx family modulate the function of nicotinic receptors (nAChRs). We report for the first time that the Lynx protein Lypd6 binds to nAChRs in human brain extracts, and that recombinant Lypd6 decreases nicotine‐induced ERK phosphorylation and attenuates nicotine‐induced hippocampal inward currents. Our findings suggest that Lypd6 is a versatile inhibitor of cholinergic signaling in the brain.

**Abbreviations used**
ACSF: artificial cerebrospinal fluid
FC: frontal cortex
GPI: glycosylphosphatidylinositol
HIP: Hippocampus
KO: knockout
WT: wild‐type

Members of the Ly‐6/neurotoxin (Lynx) superfamily have been used for decades as lymphocyte differentiation markers (Bamezai 2004). More recent research has shown that several Lynx proteins are expressed in the brain [reviewed in (Miwa *et al*. 2012)]. Lynx proteins are characterized by a three‐dimensional consensus motif, termed the three‐finger fold, that mediates protein–protein binding (Tsetlin 1999), and most Lynx proteins are attached on the cell membrane through a glycosylphosphatidylinositol (GPI) anchor (Adermann *et al*. 1999). The Lynx proteins, Lynx1 and Lynx2, have been shown to form stable complexes with, and negatively modulate the response of, α7 and α4β2 nicotinic acetylcholine receptors (nAChRs) (Miwa *et al*. 2006; Tekinay *et al*. 2009; Fu *et al*. 2012). Furthermore, Lynx1 knockout mice exhibit improved associative learning in a fear‐conditioning paradigm (Miwa *et al*. 2006), and it was recently shown that Lynx1, acting through nAChRs, is critical for the loss of synaptic plasticity in the visual cortex that occurs in adulthood (Morishita *et al*. 2010). These results suggest that Lynx1 can bind to and modulate nAChR function in the brain, and that this has important consequences for synaptic plasticity and memory [reviewed in (Miwa *et al*. 2011, 2012; Thomsen and Mikkelsen 2012;Thomsen *et al*. 2016)]. However, it is currently not known whether this regulation of the cholinergic system is unique to Lynx1 or whether it extends to other members of the Lynx family.

LY6/PLAUR domain‐containing 6 (Lypd6) is a Lynx protein that is expressed in the human and rodent nervous system (Darvas *et al*. 2009; Zhang *et al*. 2010). In the mouse visual cortex, Lypd6 and Lynx1 are mainly expressed in GABAergic interneurons, where they exhibit complementary expression patterns; the former is exclusively expressed in somatostatin and the latter is restricted in parvalbumin interneurons (Demars and Morishita 2014). This implies that Lypd6 may be involved in modulation of nAChRs in neuronal circuits distinct from Lynx1. Lypd6 is an important regulator of embryogenesis in zebrafish through its enhancement of Wnt/β‐catenin signaling (Özhan *et al*. 2013), and Lypd6 knockdown in mice results in small litter size and lack of ability to procreate, suggesting impaired germ cell or embryonic development (Darvas *et al*. 2009). In humans, two distinct microduplications of the 2q23.1 chromosomal region containing the Lypd6 gene resulted in severe intellectual disability and autistic features (Chung *et al*. 2012). Transgenic over‐expression of Lypd6 in mice increases the Ca^2+^ component of nicotine‐induced currents in dissociated trigeminal ganglion neurons and results in behaviors indicative of an increased cholinergic tone, such as locomotor arousal, hypoalgesia, and pre‐pulse inhibition of the acoustic startle response (Darvas *et al*. 2009). This invites the possibility that Lypd6, like Lynx1 and Lynx2, modulates nAChR function by binding to nAChRs. However, to the best of our knowledge, there is no report of interactions of Lynx proteins with native nAChRs in the human brain.

Nicotine, the major pharmacologically active chemical found in tobacco smoke, is another important regulator of the cholinergic system through its modulation of nAChRs in animals and humans. Pre‐ or perinatal exposure to smoking or nicotine alone can produce long‐term alterations in several transmitter systems including the glutamatergic, serotonergic, and dopaminergic (Wang *et al*. 2011; Muneoka *et al*. 1997) and is associated with development of anxiety, attention deficit hyperactivity disorder, memory deficits, and other cognitive deficits [reviewed in (Blood‐Siegfried and Rende 2010; Abbott and Winzer‐Serhan 2012; Clifford *et al*. 2012)].

Here, we report for the first time a direct interaction of Lypd6 with native human nAChRs from fresh human temporal cortical extracts and we identify Lypd6 as an inhibitor of nAChR function in rat hippocampal slices and PC12 cells. Since Lypd6 and nicotine both modulate nAChRs and also regulate developmental processes, we further sought to examine a possible interaction between the two in a developmental model of nicotine exposure, and show that nicotine administration to pregnant rats alters Lypd6 protein levels in the brain of the adult offspring.

## Materials and methods

### Human tissue and animals

Human temporal neocortical tissue was obtained from an anterior temporal lobectomy performed in a patient (female, age 58) with medically intractable temporal lobe epilepsy with hippocampal onset. Written informed consent was obtained before surgery. The study was approved by the Ethical Committee in the Capital Region of Denmark (H‐2‐2011‐104) and performed in accordance with the Declaration of Helsinki. The tissue was immediately frozen on dry ice and stored at −80°C until use.

Animal use for brain slice electrophysiology studies (Harlan Mice laboratories, Denmark) was permitted by the Animal Welfare Committee, appointed by the Danish Ministry of Justice and all experiments were in accordance with the institutional guidelines (The European Communities Council Directive of 24 November 1986 (86/609/EEC) and with Danish laws regulating experiments on animals.

Pregnant dams and adult male Sprague–Dawley (SD) rats (200–250 g) were obtained from Charles River Laboratories (Germany), and housed individually or two per cage, respectively. For the experiment with perinatal nicotine exposure, timed‐pregnant SD rats were obtained from Harlan Laboratories (Frederick, MD, USA). Pups were weaned at post‐natal day (PND) 21. Only male pups were analyzed in this study. Animal procedures were conducted with approval of the Howard University Animal Care and Use Committee (HU‐IACUC) adhering to the guidelines set forth by the National Research Council, or in accordance with the Danish National Guide for Care and Use of Laboratory animals and the European Communities Council Directive of 24 November 1986 (86/609/EEC).

Mice deficient for the α7 or β2 nAChR subunit (C57BL/6J background), and wild‐type (WT) littermates were purchased from The Jackson Laboratories and bred at Virginia Commonwealth University. Mice deficient for the β4 nAChR subunit were obtained from Baylor University School of Medicine (Xu *et al*. 1999), and subsequently maintained at the University of Colorado. Brains from 8 to 12 weeks old male or female homozygous knockout (KO) and age‐ and sex‐matched wild‐type (WT) mice were used.

Animals were kept on a 12/12 h light/dark cycle provided with standard rodent diet and water *ad libitum*. The male animals were acclimatized for a minimum of 7 days and the dams for about 24 h after arrival before experiments began.

### Developmental, tissue, cellular, and subcellular distribution of Lypd6

Tissue cross‐linking using the cell‐impermeable cross‐linking agent bis(sulfosuccinimidyl) suberate (BS^3^, Pierce Biotechnology), fractionation into crude synaptosomes or soluble and membrane fractions, culturing of neuronal and astro‐ and microglia cultures, as well as the developmental study and sampling of cerebrospinal fluid has been described previously for the same samples that are being used in this study (Thomsen *et al*. 2014).

### mRNA extraction and qPCR

Total RNA was isolated using Trizol Reagent (Sigma‐Aldrich, St. Louis, MO, USA) according to the manufacturer's instructions. RNA samples were dissolved in RNase‐free water and the RNA content was quantified using a Nanodrop ND‐1000 Spectrophotometer (Nanodrop Technologies, Wilmington, DE, USA). Samples were diluted with RNase‐free water to equal RNA concentrations and reverse transcribed into single‐stranded cDNA with Invitrogen^TM^ SuperScript^®^ III First‐Strand Synthesis system (Thermo Fisher Scientific Inc., Waltham, MA, USA) according to the manufacturer's directions using 1.25 μM oligo (dT)_20_ primers, 5 mM MgCl_2_, and 2 units RNase inhibitor. Real‐time qPCR reactions were performed in a total volume of 20 μL, containing 5 μL sample cDNA, 1× Precision Plus qPCR Master mix, premixed with SYBR‐green (Primerdesign, Southampton, UK), and 16 pmol of the forward and reverse primers (DNA technology, Aarhus, Denmark). PCR was performed on a Light Cycler^®^ 480 Real‐Time PCR System (Roche, Indianapolis, IN, USA) with a 2‐min preincubation at 95°C followed by 40 cycles of 15 s at 95°C, 10 s at 60°C, and 10 s at 72°C. The primer pair for Lypd6 was (written 5′‐3′): GCTACAAGATCTGCACCTCC and GCAAATGTGGCATCAGTGTC. Primer pairs were validated using serially diluted cDNA to establish a standard curve and by confirming the existence of a single product on a gel at the correct molecular weight. Quantification of mRNA expression was performed according to the comparative C_T_ method as described by Schmittgen and Livak (2008). For each sample, the amount of target mRNA was normalized to the mean of the PND60 group.

### Affinity purification

Recombinant human GST‐tagged Lypd6 produced in E.coli (Cusabio, Wuhan, China) and dissolved to 1 mg/mL in phosphate‐buffered saline (PBS), pH 7.4 was coupled to PureProteome^™^ NHS Flexibind magnetic beads (Millipore, Billerica, MA, USA) in a ratio of 1 : 2 (vol/vol) using the manufacturer's instructions. Successful coupling was confirmed by subsequent protein determination, showing a substantial decrease in the protein content of the Lypd6 solution. Another batch of beads without Lypd6 in the PBS was processed in parallel as a negative control. The beads were incubated in 0.1% bovine serum albumin in PBS, pH 7.4 for 1 h at 4°C prior to use.

Approximately, 100 mg human temporal neocortical tissue was lysed in 1 mL lysis buffer [50 mM Tris, 50 mM NaCl, 5 mM EDTA, 5 mM EGTA, 10 μL/mL protease inhibitor cocktail (Sigma‐Aldrich, Brøndby, Denmark], pH 7.5) using a PT1200C polytron blender (Kinematica, Luzern, Switzerland) for 20 s. The lysate was centrifuged for 30 min at 160 000 *g* at 20–22°C using an air‐driven ultracentrifuge (Airfuge^®^, Copenhagen, Denmark), and the supernatant was discarded. The pellet was resuspended in 1 mL lysis buffer containing 2% Triton X‐100 by blending for 20 s and incubated for 2 h at 4°C on a rotor (15 rpm). Thereafter, the sample was centrifuged as above and the resulting supernatant (input) was used for affinity purification. Total protein content was determined using the Pierce 660 nm Protein Assay (Thermo scientific, Rockford, IL, USA) and 700–1000 μg protein was incubated with 50 μL magnetic beads in a total volume of 1500 μL lysis buffer for 18–22 h at 4°C on a rotor (15 rpm). For experiments including α‐bungarotoxin (α‐BTX, Tocris Bioscience, Bristol, UK), the tissue was lysed in 10 ml lysis buffer and centrifugation was performed in 4°C. A quantity of 100 nM α‐BTX (final concentration) or vehicle (PBS) was added to the tissue extracts followed by incubation for 30 min on ice before addition of beads.

Subsequently, a sample of the remaining homogenate after affinity purification was taken (output) and the beads were washed twice in 1 M NaCl, 8 mM Na_2_HPO_4,_ 2 mM NaH_2_PO_4_, 0.5% Triton X‐100, pH 7.5, and thrice in 0.1 M NaCl, 8 mM Na_2_HPO_4,_ 2 mM NaH_2_PO_4_, 0.5% Triton X‐100, pH 7.5, and immediately processed for western blotting.

### PC12 cell culture and ERK phosphorylation assay

PC12 cells were maintained in 75 cm^2^ flasks coated with 5 μg/mL poly‐L‐lysine (Sigma‐Aldrich), in Dulbecco's modified Eagle medium (Gibco Life Technologies, Thermo Fisher Scientific, Waltham, MA, USA) supplemented with 10% heat‐inactivated horse serum, 5% fetal bovine serum, 25 U/mL penicillin, 25 μg/mL streptomycin, 1 mM sodium pyruvate, and 2 mM glutamine at 37°C in a humidified incubator with 5% CO_2_. Cells were subcultured every 3–4 days by detachment with 0.25% trypsin in EDTA solution (Gibco Life Technologies) and re‐seeded at 15% confluence.

For the extracellular signal‐regulated kinase (ERK) phosphorylation assay, cells were seeded in 24‐well plates at 12 × 10^4^ cells/cm^2^, 24 h prior to the experiment. On the day of the experiment, cells were incubated for 10 min with recombinant human Lypd6 protein or α‐conotoxins PIA, MII, or AuIB (Tocris Bioscience) diluted in Dulbecco's modified Eagle medium, followed by 5‐min stimulation with 25 μΜ nicotine (Sigma‐Aldrich). Thereafter, cells were lysed in 100 μL ice cold lysis buffer/well (100 mM NaCl, 25 mM EDTA, 10 mM Tris, 4 mM Na_3_VO_4_, 1 mM NaF, and 1% (v/v) Triton X‐100, 1% (v/v) NP‐40, 1 μL/mL protease inhibitor cocktail (Sigma‐Aldrich), pH 7.4). Ensure complete lysis, lysates were then placed in −80°C for 15 min, thawed, and sonicated for 5 s on ice. Lysates were stored at −80°C until use.

### Nicotine administration

Nicotine was administered to pregnant rats through a mini‐osmotic infusion pump (Model #2006, Alzet, Cupertino, CA, USA) surgically implanted between the scapulae at gestational day 7, as previously described (Wang and Gondré‐Lewis 2013; Wang *et al*. 2011). Nicotine hydrogen tartrate (Sigma‐Aldrich, St Louis, MO, USA) was prepared fresh on the day of pump implantation and delivered to dams at a rate of 4 mg/kg/day (free base weight) from pregnancy until pups reached PND21. At PND21, the pups were weaned and allowed to reach adulthood (PND60). Hippocampus (HIP) and frontal cortex (FC) from male pups were microdissected and stored at −80°C for subsequent experimentation. Frontal cortical and hippocampal tissue from the left hemisphere were lysed for western blotting, and hippocampal tissue from the right hemisphere was used for BS^3^ cross‐linking and synaptosome preparations as mentioned above. To study the effect of nicotine when administered directly to young and adult rats, 0.4 mg/kg nicotine (free base weight) or vehicle (0.9% saline) was administered subcutaneously (s.c.) twice daily for 7 days (PND8–14 or 54–60) and the FC and HIP were dissected 4 h after the last administration.

### Western blotting

Total protein content was measured using a DC Protein Assay Kit (Bio‐Rad, Hercules, CA, USA). Equal amounts of lysates were then diluted in a loading buffer (final concentration: 60 mM Tris, 10% (v/v) glycerol, 5% (v/v) mercaptoethanol, 2% (w/v) sodium dodecyl sulfate, 0.025% (w/v) bromophenol blue, pH 6.8), incubated for 5 min at 95°C, submitted to gel electrophoresis using AnykD^™^ gels (Bio‐Rad), and blotted onto polyvinylidene difluoride membranes (Bio‐Rad). Membranes were washed in tris‐buffered saline with 0.1% Tween 20 (TBS‐T) and blocked in TBS containing 5% (w/v) dry milk powder, which was also used for antibody incubations. Incubation in primary antisera against Lypd6 (1 : 1000, #ARP53451_P050, Aviva Systems Biology, San Diego, CA, USA), Lynx1 (1 : 1000, #sc‐23060, Santa Cruz Biotechnology, Heidelberg, Germany), Ly6H (1 : 1000, #H00004062‐M01, Novus Biologicals, Cambridge, UK), α3, α4, α5, α6 (1 : 100 #sc‐1771, sc‐5591, sc‐28795, sc‐27292 Santa Cruz Biotechnology) for which the selectivity has been previously characterized (Guan *et al*. 2001; Lang *et al*. 2003; Kabbani *et al*. 2007; Yu *et al*. 2007), β2 (1 : 1000, provided by Dr Cecilia Gotti) and α7, β4 (1 : 1000 #ab23832 and 1 : 100 #ab156213 Abcam, Cambridge, UK) which we have validated in this study (Fig. S3), Actin (1 : 10 000, #A5060, Sigma‐Aldrich), GluR2 (1 : 200, #MABN71, Millipore), PSD‐95 (1 : 2000, #ab9708, Millipore), phosphorylated ERK1/2 (1 : 4000 #9101 Cell Signaling, Leiden, the Netherlands), or ERK1 (1 : 4000, #610031, BD Transduction Laboratories, Franklin Lakes, NJ, USA) was done overnight at 4°C on parafilm in a humidified container, followed by 3 × 10 min washes in TBS‐T and 1 h incubation at 20–22°C in horseradish peroxidase‐conjugated secondary antibody (1 : 2000, Dako, Glostrup, Denmark). After thorough washing in TBS‐T, enhanced chemiluminescence western blotting detection reagents (Western Lightning^®^ ECL Pro, Perkin Elmer, Waltham, MA, USA) were used for signal detection and protein bands were visualized using an automated film developer (Colenta Labortechnik, Wiener Neustadt, Austria). Mean optical densities of bands were measured and their corresponding background measurement subtracted.

### Brain slice preparation and electrophysiology

Patch clamp, whole‐cell recordings were made from hippocampal CA1 interneurons in rat brain slices to determine the effects of Lypd6 on membrane current responses to nAChR agonists. Preparation of brain slices containing hippocampal CA1 neurons was conducted essentially as previously described (Soni and Kohlmeier 2016). Briefly, 12‐ to 15‐day‐old Naval Medical Research Institute (NMRI) wild‐type mice of either sex were anesthetized with isoflurane, decapitated, and the brain was rapidly removed and placed into an ice‐cold (0–4°C) artificial cerebrospinal fluid (ACSF) which contained (in mM): 124 NaCl, 5 KCl, 1.2 Na_2_HPO_4_, 2.7 CaCl_2_, 1.2 MgSO_4_, 10 glucose, and 2.6 NaHCO_3_, bubbled with 95% O_2_/5% CO_2_ resulting in a pH of 7.4. The block of the brain containing the HIP was sectioned into 250‐μm thick coronal slices using a vibrotome (Leica VT 1200S, Leica, Germany) after vertical deflection was minimized. Thereafter, slices containing the HIP were incubated in ACSF at 37°C for ~20 min followed by maintenance at 20–25°C for 1 h to reach equilibration.

All recordings were conducted in a submersion recording chamber at 20–25°C. Whole‐cell recordings were obtained from CA1 interneurons, identified visually using an upright microscope (Olympus BX51WI, Hamburg, Germany). Recording pipettes were made from borosilicate glass capillary tubing (1.5 mm O.D), pulled on a horizontal puller (Sutter Instruments, P‐95) and filled with internal patch solution (resistance of ~6 MΏ) containing: (in mM) 144 K gluconate, 3 MgCl_2_, 10 HEPES, 0.3 NaGTP, and 4 Na_2_ATP (295–300 mOsM). Membrane currents were recorded using an Axopatch‐200B amplifier (Axon Instruments) and were filtered at 2 KHz (low‐pass Bessel filter) at the amplifier output and digitized at 20 KHz, with subsequent analysis of current amplitudes performed with Clampex 10.3 (Molecular Devices, Sunnyvale, CA, USA).

Membrane responses induced by the activation of nAChRs on CA1 interneurons were recorded following application of nicotine (100 μM, Sigma, St. Louis, MO, USA), or the nAChR agonist, Dimethylphenylpiperazinium (DMPP) (50 mM, Sigma), in the absence or presence of human recombinant Lypd6 protein (6 μM, Cusabio). All agonists were applied from borosilicate micropipettes ( ~2 MΩ) using pressure delivered via a picospritzer III (Parker Hannifin Corporation, Mayfield Heights, OH, USA) at a distance of ≤ 100 μm from the recorded soma, with a constant pressure maintained at 10 psi for 10–50 ms. Lypd6 was also applied using a borosilicate micropipette ( ~500 KΩ) using pressure delivered via a picospritzer III at a distance of ~200 μm from recorded somas, with a constant pressure maintained at 10 psi for a duration of 4–5 s. During Lypd6 application, bath perfusion of ACSF was stopped for 3–4 min in order to maximize the diffusion of Lypd6 into the slice. To check the specificity of Lypd6‐dependent effects on nAChR responses, the order of drug application was reversed in a few recordings, such that slices were exposed to Lypd6, then nAChR agonists were applied.

### Statistical analysis

Data were analyzed using two‐way anova with Sidak‐corrected multiple comparisons or Bonferroni‐corrected ratio paired *t*‐tests. For the electrophysiological recordings, data were analyzed using unpaired or paired *t*‐tests. The statistical calculations were performed using GraphPad Prism version 6 for Windows (GraphPad Software, San Diego, CA, USA). All data are presented as mean ± standard error of the mean, and a *p*‐value of < 0.05 was considered statistically significant.

## Results

### Distribution and developmental expression of Lypd6

**Figure 1:**
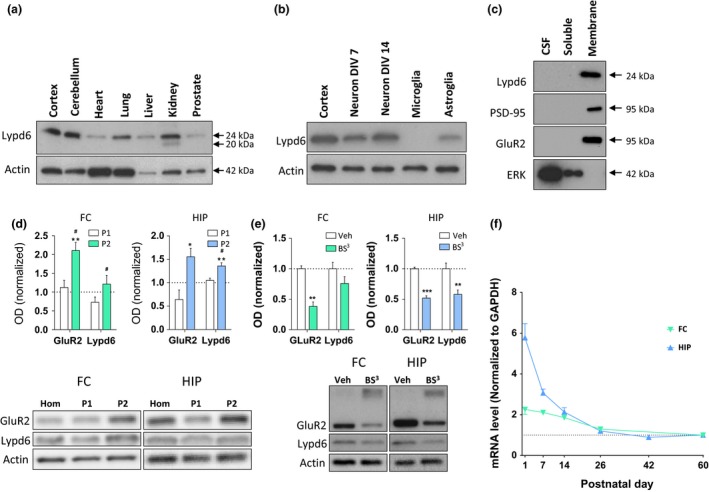
Distribution and developmental expression of Lypd6. (a–e) Representative images and analysis of western blots showing Lypd6 protein levels in: (a) different organs of adult male rats (*n* = 2) in relation to actin, (b) cortical tissue from adult male rats, rat cortical primary neurons cultured for 7 or 14 days *in vitro* (neuron DIV7 and 14, respectively), rat primary microglia cultured for 15 days *in vitro*, and rat primary astroglia cultured for 18 days *in vitro* (*n *=* *3) in relation to actin, (c) cerebrospinal fluid (CSF), and soluble and membrane fractions of cortical tissue from adult male rats. The membrane‐bound proteins, PSD‐95 and GluR2, and the soluble protein extracellular signal‐regulated kinase 1 (ERK) are used as controls for effective fractionation (*n* = 4), (d) FC and HIP tissue fractionated using differential centrifugation into a pellet 1 (P1, nuclear) and pellet 2 (P2, crude synaptosome) fraction. Data are normalized to actin levels and the level of the non‐fractionated homogenate (Hom) was set to 1 (dashed line). *p <* *0.05, **p <* *0.01 indicates statistical difference between non‐fractionated homogenate and the P2 fraction, ^#^ *p *<* *0.05 indicates statistical difference between the P1 and P2 fractions in a Bonferroni‐corrected ratio paired *t*‐test (*n *=* *5), (e) minced tissue extracts from the FC or HIP of adult male rats cross‐linked with bis(sulfosuccinimidyl) suberate (BS ^3^) or vehicle (Veh). The staining for GluR2 shows that cross‐linking with BS ^3^ reduces the intensity of the GluR2 band at 95 kDa, and produces a smear of high‐molecular weight cross‐linked proteins. Data are normalized to actin levels and the vehicle group was set to 1. **p <* *0.01, ***p <* *0.001 indicates statistical difference between vehicle‐ and BS ^3^‐treated tissue in a Bonferroni‐corrected ratio paired *t*‐test (*n* = 9–11). (f) Lypd6 mRNA levels in frontal cortex (FC) and hippocampus (HIP) from male rats killed at post‐natal day 1, 7, 14, 26, 42, or 60 (*n* = 5–6). The values were normalized to GAPDH levels.

To characterize the expression pattern of Lypd6, we analyzed Lypd6 protein levels in different organs of adult male rats using western blotting. A band corresponding to Lypd6 protein was detected in all organs analyzed with high levels in the cortex and cerebellum of the brain, moderate levels in the lung, kidney, and liver, and low levels in the heart and prostate (Fig. 1a). In the kidney, the Lypd6 antibody detected an additional lower molecular weight band.

We further detected high levels of endogenous Lypd6 protein in rat primary cortical neurons cultured to 14 DIV (Fig. 1b). Cortical neurons cultured to 7 DIV and astroglia cultured to 18 DIV displayed moderate staining, whereas Lypd6 was not detectable in primary microglia cultured to 15 DIV.

Previous studies have shown that Lynx proteins are not only expressed as GPI‐anchored membrane proteins but may also exist as soluble proteins (Adermann *et al*. 1999; Bamezai 2004). Fractionation of rat cortical tissue into soluble and membrane fractions by ultracentrifugation and subsequent western blotting alongside samples of rat cerebrospinal fluid (CSF) revealed that Lypd6 was detectable in the membrane fraction, but not in the soluble fraction or in CSF samples (Fig. 1c). To validate the separation, we demonstrated that the membrane‐associated scaffolding protein, post‐synaptic density 95 (PSD‐95), and the α‐amino‐3‐hydroxy‐5‐methylisoxazole‐4‐propionate receptor subunit GluR2 were only present in the membrane fraction, whereas the soluble protein ERK1 was only present in the soluble fraction.

To determine the subcellular localization of Lypd6, tissue homogenates from the FC and HIP were fractionated into nuclear (P1) and synaptosomal (P2) fractions (Fig. 1d). There was a significantly higher level of Lypd6 in the HIP synaptosomal fraction compared to the total homogenate (*p *<* *0.01). Additionally, comparing the synaptosomal and nuclear/endosomal fractions revealed a significantly higher level of Lypd6 in synaptosomes in both the FC and HIP (both *p *<* *0.05). GluR2 was used as a positive control as it is known to be located in the synaptosomal fraction (Srivastava *et al*. 1998).

To determine the extent of surface expression of Lypd6, protein levels in tissue extracts from the FC or HIP of adult rats, cross‐linked using the cell‐impermeable protein cross‐linking agent BS^3^, were compared with untreated extracts (Fig. 1e), and the GluR2 receptor subunit was used as a positive control and reference value (Grosshans *et al*. 2002; Mielke and Mealing 2009; Carino *et al*. 2012). In the HIP, the Lypd6 signal was reduced by 42 ± 0.07% (mean ± SEM, *p *<* *0.01) compared to non‐cross‐linked tissue extracts, corresponding to 87% of the signal reduction seen with GluR2, showing that Lypd6 is predominantly present on the cell surface. In the FC, the Lypd6 signal was reduced by 24 ± 0.11%, but was not significantly different from vehicle controls.

We finally studied the expression pattern of Lypd6 during development. We measured Lypd6 mRNA levels in the FC and HIP of rats at PND1, 7, 14, 26, 42, and 60 (Fig. 1f). Lypd6 mRNA was present in the FC at birth, but decreased to PND26 with a slower decrease from PND26 until PND60. In the hippocampal formation, Lypd6 mRNA was detected at high levels at birth and the levels decreased to near adult levels within the first three post‐natal weeks.

Taken together, these data indicate that Lypd6 is a membrane‐bound protein, predominantly expressed in the rodent brain and highly enriched at the synaptic loci.

### Lypd6 binds to multiple nAChRs in human brain extracts

**Figure 2:**
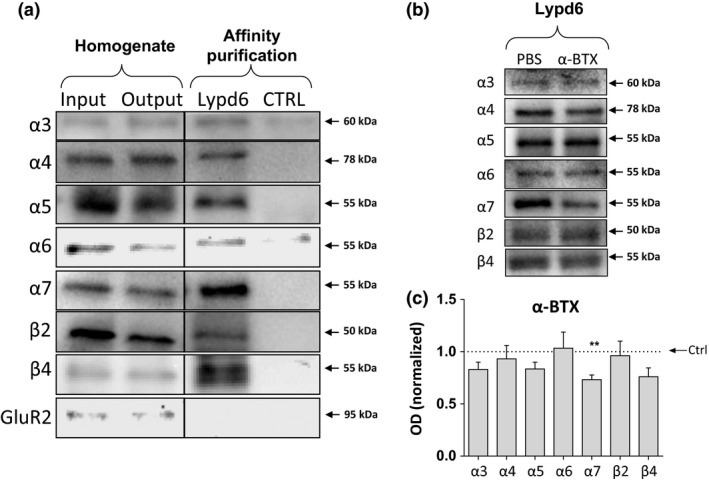
Lypd6 binds multiple nAChR subunits in the human brain. (a) Cortical homogenates from human temporal cortex (input) were affinity purified using magnetic beads covalently coupled with recombinant human Lypd6 (Lypd6) or non‐coupled beads (ctrl). Also shown is the remaining homogenate after affinity purification (output). Samples were submitted to gel electrophoresis and western blotting followed by detection of nAChR subunits and the GluR2 AMPA receptor subunit. (b) Representative images of western blots of Lypd6 affinity purification of human temporal cortex homogenates pre‐incubated with 100 nM α‐BTX or vehicle [phosphate‐buffered saline (PBS)] for 30 min. (c) Quantification of data represented in (b), showing that α‐BTX interferes with binding of the α7 nAChR subunit to Lypd6‐coupled beads (*n* = 5). Values are normalized to their individual vehicle and represented as mean ± SEM. **p <* *0.01 indicates statistical difference from affinity purification in the absence of α‐BTX in a ratio paired *t*‐test.

Members of the Lynx family have been demonstrated to bind to nAChRs altering their function (Ibañez‐Tallon *et al*. 2002; Tekinay *et al*. 2009; Jensen *et al*. 2015). To test the binding of Lypd6 for nAChR subunits, we performed affinity purification using bead‐coupled recombinant human Lypd6 as bait. In extracts from human temporal cortex, Lypd6 was co‐purified with α3, α4, α5, α6, α7, β2, and β4 nAChR subunits (Fig. 2a), but not with GluR2. None of the nAChR subunits were detected when the affinity purification was performed using non‐coupled beads, confirming that co‐purification of nAChR subunits was attributable to a specific interaction with Lypd6. The selectivity of α7, β2, and β4 nAChR subunit antibodies used was verified by performing affinity purification with human recombinant Lypd6‐coupled beads on α7, β2, and β4 KO mice cortical tissue, showing that each of the subunits was only detected in the corresponding WT, but not KO homogenates (Fig. S3). Although quantitative assessment is not possible because of a low sample number, visual comparison of the input and output homogenates from the affinity purification suggest a reduction in band intensity for α4, α6, and α7, indicating that a detectable proportion of these nAChR subunits have been co‐purified by Lypd6.

Pre‐incubation of human temporal cortical extracts with the α7‐specific orthosteric antagonist α‐BTX (100 nM) significantly reduced the amount of the α7 nAChR subunit (26.8 ± 0.04%, *p *<* *0.01) isolated during the Lypd6 affinity purification compared to pre‐incubation with PBS, whereas it did not significantly affect the amount of α3, α4, α5, α6, β2, or β4 purified (Fig. 2b and c). These results suggest that Lypd6 is able to interact with several nAChR subunits in the human cortex, and that Lypd6 competes with α‐BTX for binding to the α7 nAChR.

### Lypd6 regulates nicotine‐induced ERK phosphorylation

**Figure 3:**
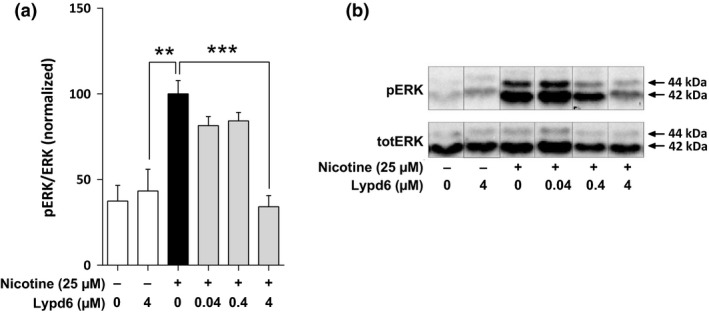
Lypd6 inhibits nicotine‐induced ERK phosphorylation. (a) Pre‐incubation for 10 min with 4 μM recombinant human Lypd6 reduces nicotine‐induced ERK phosphorylation in PC12 cells, after stimulation with 25 μM nicotine for 5 min. (b) Representative images of a western blot membrane summarized in (a), where the phosphorylated isoforms p44 and p42 of the MAP kinase ERK (ERK1/2) are represented in the top panel (pERK), whereas the total amount of unphosphorylated ERK 1/2 is indicated in the bottom panel (totERK). **p < 0.01, ***p < 0.001 indicates statistical difference from the nicotine group in a one‐way ANOVA test.

To further assess the functional effect of Lypd6 binding to nAChRs, we used rat pheochromocytoma PC12 cells that natively express nAChRs (Nakayama *et al*. 2006). Stimulation with 25 μM nicotine significantly increased phosphorylation of the MAP kinase ERK in PC12 cells. This increase was completely inhibited by pre‐incubation with 4 μM recombinant human Lypd6 (Fig. 3). Lower concentrations of recombinant Lypd6 (0.04 and 0.4 μM) also slightly reduced ERK phosphorylation, but this did not reach significance. Lypd6 exposure alone did not affect basal levels of ERK phosphorylation. We have previously shown that the effect of nicotine on ERK phosphorylation in the PC12 cell line we used here is dependent on α3β4‐containing nAChRs (Arvaniti *et al*. 2015).

### Lypd6 attenuates nicotine‐induced hippocampal inward currents

**Figure 4:**
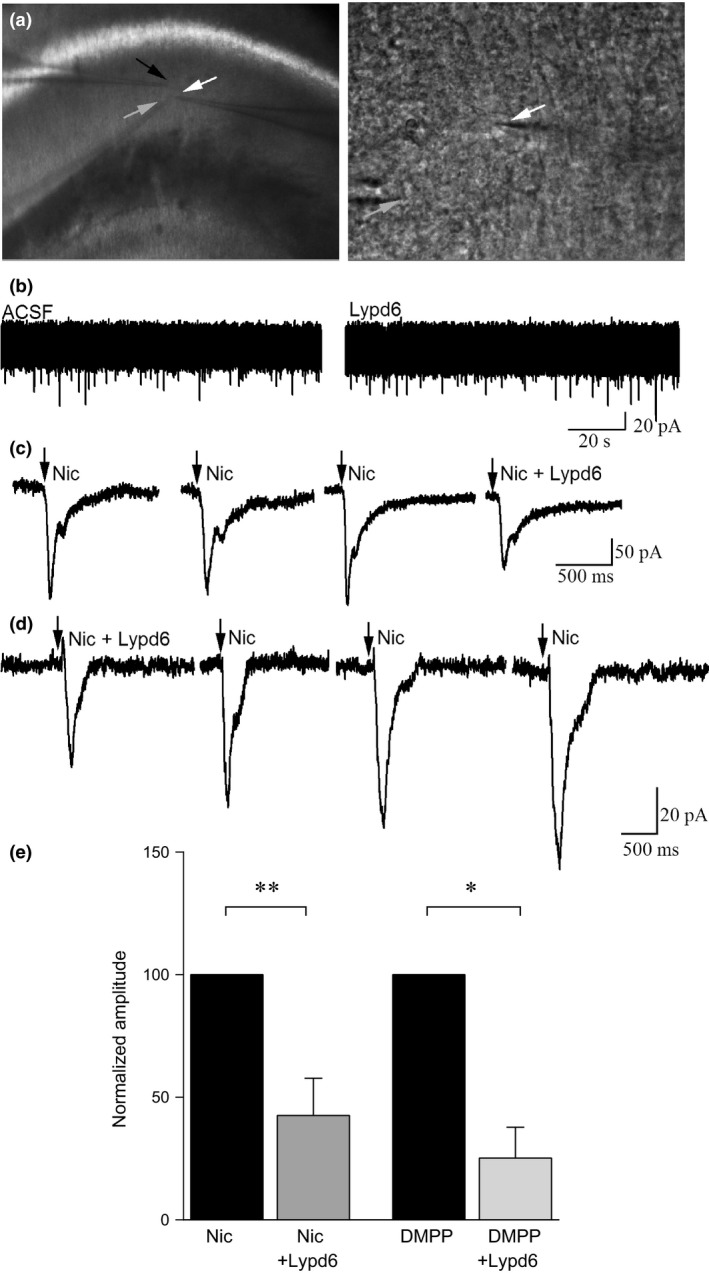
Lypd6 reversibly attenuates nAChR‐induced membrane responses in CA1 hippocampal interneurons. (a) Bright field image of the stratum radiatum of the CA1 hippocampal region where recordings were conducted (white arrow indicates recorded cell). Lypd6 (6 μM) was applied via a 2nd patch pipette (black arrow) close to the recorded cell and nicotine was applied via a 3rd pipette (gray arrow). Higher magnification image of a CA1 interneuron recorded for this study (white arrow). The electrode delivering nicotine is also visible (gray arrow). (b) Whole‐cell voltage clamp recordings from a CA1 interneuron showing no effect on induction of inward current by application of Lypd6. (c) An example of whole‐cell voltage clamp recordings from a CA1 interneuron showing induction of inward current by application of nicotine (arrow) and attenuation of this current when applied in the presence of Lypd6 (Nic + Lypd6). Nicotine responses were not attenuated with a 3‐min interval between applications, indicating that reductions induced in the presence of Lypd6 were not because of cell run down, but were specific to the presence of the protein. (d) An example of whole‐cell voltage clamp recordings from a CA1 interneuron, pre‐incubated in Lypd6 for 5 min before first application of nicotine, showing that the effect of Lypd6 is lost upon wash‐out. The recording examples shown in (c) and (d) are not representing the mean values, but were rather chosen as high‐amplitude samples to clearly demonstrate the inhibitory effect of Lypd6. (e) Graphs of mean responses to nicotine (n_cells_ = 5) and to the non‐subtype‐specific nAChR agonist DMPP (n_cells_ = 3) from the population of cells recorded, indicating that when Lypd6 is present, the amplitude of inward currents induced by activation of nAChRs is significantly reduced. Data are displayed as each cell's response to the agonist in the presence of Lypd6 normalized to the same cell's response before the addition of Lypd6. Values are represented as mean ± SEM. *p <* *0.05, **p <* *0.01, indicates statistical difference between groups in a paired *t*‐test.

Whole‐cell recordings were obtained from visually identified hippocampal CA1 interneurons (Freund and Buzsáki 1996; Alkondon and Albuquerque 2001) of stratum oriens and stratum radiatum, where these interneurons have horizontally oriented spindle‐shaped cell bodies and this feature is characteristically distinct from pyramidal neurons (Fig. 4a). Hippocampal interneurons where chosen because of the abundant expression of the α7 nAChR subtype (Frazier *et al*. 1998; Buhler and Dunwiddie 2001, 2002).

Lypd6, when applied alone, did not induce an outward or inward membrane current (Fig. 4b). When held at −60 mV, nicotine application (100 μM, 10–50 ms duration) resulted in an inward current of 67.2 ± 22.0 pA, which was repeatable with an interval of at least 3 min between nicotine applications (*n* = 5, Fig. 4c and d). In the presence of Lypd6 (6 μM), the average reduction in nicotine‐induced inward currents in each individual neuron was 57.4 ± 15.2% (mean ± SEM, paired *t*‐test, *p *<* *0.01, *n*_cells_ = 5, Fig. 4e). Similarly, the non‐specific nAChR agonist, DMPP (50 μM) induced an average inward current of 29.7 ± 7.2 pA, which was attenuated by 74.8 ± 12.6% when DMPP was applied in the presence of Lypd6 (n_cells_ = 3, *t*‐test, *p *<* *0.05; Fig. 4e).

We further examined whether Lypd6‐mediated attenuation of inward currents was because of order effects, or run down of nAChR responses because of cellular exhaustion. After pre‐incubation with Lypd6, nicotine was applied repeatedly, with a recovery time in between applications of ~ 3 min (Fig. 4d). A gradual increase in the amplitude of nicotine‐induced inward currents was noted, suggesting that the observed effect was associated only with the presence of Lypd6 and could be reversed.

### Perinatal nicotine exposure increases Lypd6 levels

**Figure 5:**
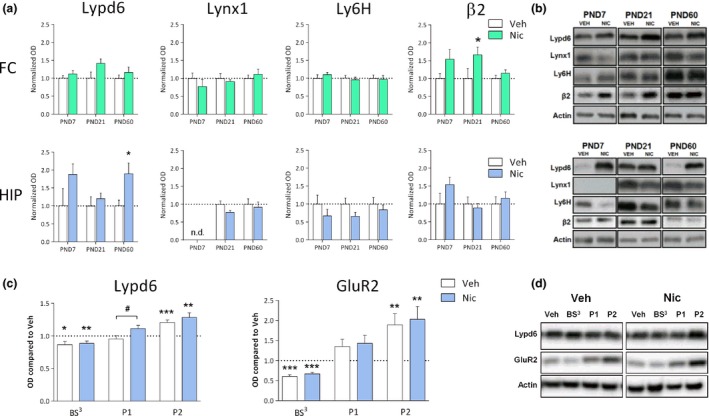
Perinatal nicotine exposure increases hippocampal Lypd6 levels. (a) Quantification of Lypd6, Lynx1, and Ly6H protein levels as well as the β2 nAChR subunit from frontal cortex (FC) and hippocampus (HIP) of male rats exposed to nicotine from embryonic day 7 to post‐natal day (PND) 21 through osmotic minipumps implanted into pregnant dams. Animals were killed at PND7 (*n* = 5), 21 (*n* = 10), or 60 (*n* = 13). Main effect of nicotine treatment in a two‐way anova with age and treatment as the fixed factors is indicated in the graphs. *p <* *0.05 indicates statistical difference in a Sidak‐corrected multiple comparisons test. (b) Representative western blot images of protein levels from FC and HIP at the different ages. (c) Quantification of Lypd6 and GluR2 protein levels in hippocampal tissue from the P60 groups described (a), showing that perinatal nicotine exposure increases hippocampal Lypd6 levels in nuclear/endosomal fractions. Tissue was fractionated using differential centrifugation into a pellet 1 (P1, nuclear) and pellet 2 (P2, crude synaptosome) fraction or cross‐linked with bis(sulfosuccinimidyl) suberate (BS ^3^). Data are normalized to actin levels and the level of the untreated, non‐fractionated homogenate was set to 1 (dashed line). *p <* *0.05, **p <* *0.01, ***p <* *0.001 indicates statistical difference from homogenate in a ratio paired *t*‐test, ^#^ *p *<* *0.05 indicates statistical difference between vehicle‐ and nicotine‐treated groups in an unpaired t‐test. (d) Representative western blot images of protein levels shown in (c).

We analyzed the protein levels of Lypd6, Lynx1, and Ly6H at PND7, 21, and 60 in the FC and HIP of male rats exposed to continuous infusion of nicotine via osmotic minipumps implanted into the pregnant dams (Fig. 5a and b).

A two‐way anova with age and treatment as the fixed factors revealed a significant main effect of nicotine treatment on Lypd6 levels in the HIP (*p *<* *0.01), but did not reach significance in the FC (*p *=* *0.07). A subsequent Sidak‐corrected multiple comparison on HIP samples showed that nicotine significantly increased Lypd6 levels at PND60 (Fig. 5a). A main effect of nicotine was also demonstrated on β2 levels in the FC (*p *<* *0.01), where a multiple comparisons test showed a significant effect of nicotine on β2 levels at PND21. Two‐way anovas on Lynx1 or Ly6H levels showed no significant main effect of nicotine.

**Figure 6:**
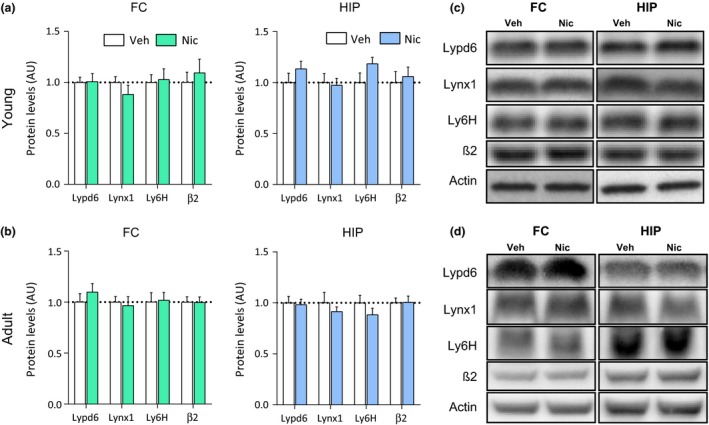
Short‐term nicotine exposure does not alter Lypd6, Lynx1, or Ly6H levels in the brain. Lypd6, Lynx1, Ly6H, and β2 nAChR subunit protein levels were analyzed in (a) frontal cortex (FC) and hippocampal (HIP) tissue from rats administered nicotine (0.4 mg/kg s.c., twice daily) or vehicle (0.9% saline) for 7 days from day 8–14 or (b) 54–60 (*n* = 8). (c and d) Representative images of western blots summarized in (a) and (b), respectively.

To further characterize the effect of nicotine on hippocampal Lypd6 levels, we demonstrate that nicotine administration significantly increased Lypd6 levels in the putative nuclear fraction (P1, *p *<* *0.05), whereas there was no difference between the groups in synaptosomal fractions (P2) or on Lypd6 surface levels, as measured using BS^3^ cross‐linking of proteins (Fig. 5c). To validate the methods, we show that GluR2 is reduced by cross‐linking and increased in synaptosomes in the two groups. Administration of nicotine (0.4 mg/kg s.c. twice daily for 7 days) to young or adult rats, did not alter protein levels of Lypd6, Lynx1, or Ly6H in the FC or HIP (Fig. 6).

**Figure 7:**
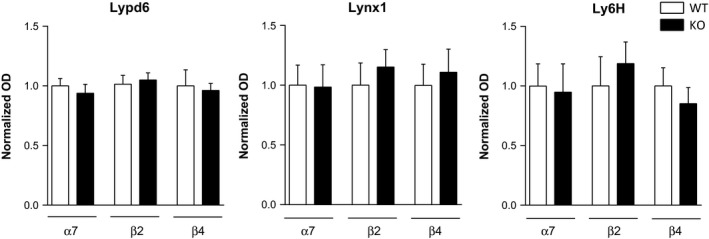
Knockout of nAChR subunits does not alter Lypd6, Lynx1, or Ly6H levels in frontal cortex. Quantification of Lypd6, Lynx1, and Ly6H protein levels from FC of mice that were either wild‐type (WT) or had a genetic deletion knockout (KO) of the β2 (*n* = 7–8), α7 (*n* = 7–8), or β4 (*n* = 4) nAChR subunit gene. Data are normalized to actin levels and the level of the respective WT was set to 1. Unpaired *t*‐tests revealed no significant differences between WT and KO mice.

Moreover, Lypd6, Lynx1, and Ly6H protein levels in FC from wild‐type and β2, α7, or β4 knockout mice were compared using unpaired t‐tests. No significant differences were found after genetic deletion of these nAChR subunits (Fig. 7).

## Discussion

Here, we show that recombinant Lypd6 binds to multiple nAChR subunits in the human brain and inhibits nicotine‐mediated activation of nAChRs *in vitro*. Furthermore, perinatal nicotine administration leads to increased hippocampal Lypd6 protein levels in adulthood. These results suggest that Lypd6 modifies cholinergic neurotransmission by a direct interaction with mature nAChRs in the brain, and that this regulation may be altered by maternal exposure to nicotine.

### Lypd6 is situated to regulate neurotransmission in the brain

Lypd6 protein was detectable in all organs examined and had a high expression in the brain. The ubiquitous expression of Lypd6 protein is in accordance with Lypd6 mRNA expression in human and mouse organs (Darvas *et al*. 2009; Zhang *et al*. 2010). In primary cultures, Lypd6 is expressed by neurons and astroglia, but not by microglia. We have previously demonstrated that the Lynx proteins, Lynx1 and Ly6H, are exclusively expressed in neurons (Thomsen *et al*. 2014), but other Lynx proteins have also been detected in glia (Cray *et al*. 1990; Rogers *et al*. 1996; Tanaka *et al*. 1997; Mildner *et al*. 2007).

Lynx proteins may exist as GPI‐anchored membrane proteins or soluble proteins (Adermann *et al*. 1999; Bamezai 2004). We detected Lypd6 in membrane fractions, but not soluble fractions or CSF samples and found that Lypd6 was predominantly localized to synaptosomes in the rat cortex, suggesting that Lypd6 is a membrane protein involved in synaptic function. This is in line with *in silico* analysis of the *Lypd6* gene in zebrafish, showing that it contains a possible GPI‐attachment site, as well as studies in both zebrafish embryos and a mammalian cell line, showing that Lypd6 is GPI‐anchored on the cell membrane (Özhan *et al*. 2013). In contrast, other members of the Lynx family such as the prostate stem cell antigen are detected only in the soluble fraction (Jensen *et al*. 2015). This discrimination may underlie differences in the mode of action, as a soluble version of the Lynx protein Lynx1 has been shown to exert a distinctive phenotype compared to the full‐length form, when expressed in a transgenic mouse model (Miwa and Walz 2012).

We further found that Lypd6 mRNA is highly expressed during early development, showing high levels in the first post‐natal days in rat FC and HIP, followed by a decrease toward adulthood. Our results imply a potential crucial role for Lypd6 in early mammalian development and support previous data, showing Lypd6 to be essential in vertebrate embryogenesis, by being involved in mesoderm and neuroectoderm patterning in zebrafish gastrulation (Özhan *et al*. 2013). In addition, the developmental expression pattern of several other Lynx proteins in the brain, such as Lynx1, Lynx2, or Ly6H, as well as the involvement of Lynx1 in the regulation of plasticity in the visual cortex further support a role of this protein family in development (Dessaud *et al*. 2006; Morishita *et al*. 2010; Thomsen *et al*. 2014).

### Lypd6 binds to and regulates nAChRs

We detected co‐purification of α3, α4, α5, α6, α7, β2, and β4 nAChR subunits by affinity pull‐down from human cortical extracts, using recombinant human Lypd6. This is the first demonstration of a direct interaction between a Lynx protein and native nAChRs in the human brain. These data indicate that Lypd6 protein is able to bind to multiple nAChR subunits in the human brain, including the clinically relevant α7 nAChR [reviewed in (Thomsen and Mikkelsen 2012)]. We have previously shown that a soluble version of Lynx1 also binds multiple nAChR subunits (Thomsen *et al*. 2014), but this broad spectrum of binding is not a feature of all Lynx proteins, as SLURP‐1 has been shown, using the same method, to bind selectively to α7 nAChRs (Lyukmanova *et al*. 2016).

To specifically investigate the interaction between Lypd6 and the human α7 nAChR, we used α‐BTX and showed that it can interfere with α7 binding to Lypd6. This suggests that Lypd6 binds orthosterically to α7 nAChRs, although we cannot exclude the possibility that binding of α‐BTX leads to conformational changes on the α7 nAChR, changing its affinity for Lypd6. If indeed Lypd6 binds on the orthosteric site, it indicates that Lypd6 could act as an antagonist, rather than an allosteric modulator, of nAChR function. In contrast, Lynx1 does not compete with α‐BTX for binding to human α7 nAChRs or with epibatidine for binding to α4β2 nAChRs, suggesting that it binds outside of the orthosteric binding site (Lyukmanova *et al*. 2011).

The direct binding between Lypd6 and nAChRs prompted us to ask whether this had an effect on nAChR function. We show that a soluble version of Lypd6 completely inhibits nicotine‐induced phosphorylation of ERK in PC12 cells and reversibly attenuates nicotine‐induced inward currents in hippocampal interneurons of rat brain slices. This is in line with previous studies, indicating a modulatory role of Lynx proteins on whole‐cell currents (Miwa *et al*. 1999; Ibañez‐Tallon *et al*. 2002; Puddifoot *et al*. 2015; Wu *et al*. 2015; Lyukmanova *et al*. 2016). Notably, Lypd6 exposure did not affect the baseline membrane current or basal levels of ERK phosphorylation, suggesting that the inhibitory effect is specifically related to nAChR signaling and that Lypd6 is not able to activate nAChRs. Activation of the ERK/MAPK pathway by phosphorylation is an important step in the formation of Long‐term potentiation (LTP) and memory in the HIP (Adams and Sweatt 2002), and nicotine‐mediated enhancement of LTP in the HIP is dependent on ERK (Welsby *et al*. 2009). In accordance with previous findings (Nakayama *et al*. 2006), we have shown that this effect of nicotine in PC12 cells is dependent on α3β4‐containing nAChRs (Arvaniti *et al*. 2015), suggesting that Lypd6 affects the function of α3β4‐containing nAChRs. This is not to say, however, that the effects of Lypd6 in the brain are restricted to α3β4‐containing nAChRs.

Our results suggest that Lypd6 can act as an endogenous orthosteric antagonist of nAChRs in the brain. This sets Lypd6 aside from other Lynx proteins, such as Lynx1 and Lynx2, which have been shown to bind to and negatively regulate the function of α7 and α4β2 nAChRs in heterologous expression systems (Ibañez‐Tallon *et al*. 2002; Tekinay *et al*. 2009), suggesting an allosteric mode of action. However, we have employed a soluble variant of Lypd6 that could possibly function differently than, or bind to sites otherwise unreachable by, endogenous GPI‐anchored Lypd6 on nAChRs, as previously speculated for the soluble Lynx1 protein (Miwa and Walz 2012).

It has previously been shown that transgenic over‐expression of Lypd6 in mice increases nicotine‐induced calcium currents and enhances behaviors associated with cholinergic neurotransmission (Darvas *et al*. 2009). Our finding that Lypd6 forms a stable complex with nAChRs suggests that these effects may be caused by a direct interaction between Lypd6 and nAChRs. The apparent discrepancy between an enhancement of calcium currents and a decrease in inward currents and phosphorylation of ERK by Lypd6 may be explained by the experimental setups. We have employed a short exposure to a soluble variant of Lypd6, which likely affects primarily surface nAChRs, and which might bind differently to nAChRs compared with endogenous Lypd6. Transgenic over‐expression of Lypd6, on the other hand, may have more complex effects, possibly involving chaperoning of nAChRs by Lypd6 in the endoplasmic reticulum, as has previously been demonstrated for the Lynx proteins Lynx1 and Ly6H (Nichols *et al*. 2014; Puddifoot *et al*. 2015). Lypd6 protein is primarily intracellular when over‐expressed in COS‐7 cells (Zhang *et al*. 2010), which is in line with a potential intracellular function of Lypd6 after transgenic expression. We demonstrate that endogenous Lypd6 is predominantly located on the cell surface in the rat HIP, whereas this localization is not as pronounced in the FC. Given the potential opposing function of surface versus intracellular Lypd6, this differential subcellular distribution of Lypd6 may have a significant impact on the function of Lypd6 in different brain regions.

### Nicotine regulates hippocampal Lypd6 levels

Since nicotine and Lypd6 both affect nAChR function as well as early development, we found it pertinent to examine whether exposure to nicotine during development affected Lypd6 protein levels in the brain. Using an exposure paradigm known to result in stable plasma levels of nicotine corresponding to that found in light to moderate smokers (Matta *et al*. 2007), we found that perinatal nicotine exposure to dams produced an almost twofold increase of Lypd6 in the HIP of the adult offspring, whereas there were no effects on the Lynx proteins Lynx1 and Ly6H. This suggests that nicotine has long‐term effects on Lypd6 expression.

Maternal smoking during or after pregnancy can have long‐term detrimental effects on the development of the offspring and lead to lifelong impairments (Blood‐Siegfried and Rende 2010; Abbott and Winzer‐Serhan 2012; Clifford *et al*. 2012). Pre‐natal nicotine exposure reduces hippocampal levels of phosphorylated ERK in rats, and this has been suggested to underlie the cognitive deficits observed after gestational nicotine exposure (Parameshwaran *et al*. 2013). We find that Lypd6 inhibits nAChR‐mediated ERK phosphorylation *in vitro* and that perinatal nicotine administration increases Lypd6 levels, suggesting that decreased ERK phosphorylation after pre‐natal nicotine administration may be caused by increased inhibition of nAChRs by Lypd6. It is therefore possible that part of the detrimental effect of nicotine on the development of the offspring is because of dysregulation of Lypd6.

We further showed that the increase in Lypd6 following nicotine exposure was primarily evident in a putative nuclear/endosomal fraction, but not in synaptosomes, suggesting that nicotine leads to a preferential increase in non‐synaptic Lypd6. If Lypd6 is able to chaperone nAChRs, nicotine administration might therefore alter this chaperoning. The effect of nicotine on Lypd6 levels was not recapitulated by nicotine administration to young or adult rats, suggesting that Lypd6 is more susceptible to regulation by nicotine during early development.

Nicotine administration up‐regulates nAChRs in the brain, including the α3β4 subtype (Govind *et al*. 2009; Mazzo *et al*. 2013). We therefore hypothesized that the effect of nicotine exposure on Lypd6 protein levels was an indirect effect caused by up‐regulation of nAChRs. However, genetic ablation of β2‐, β4‐, or α7‐containing nAChRs did not affect Lypd6 levels, suggesting that decreasing the levels of the major nAChR subtypes in the brain does not affect Lypd6 protein levels. It is, however, shown that Lynx1 interacts preferentially with α4/α4 dimers in α4β2 nAChRs (Nichols *et al*. 2014), and if Lypd6 preferentially binds to α/α interfaces as well, we cannot exclude a potential effect of nicotine via α‐subunit up‐regulation, other than α7. In a further attempt to see whether cholinergic tone or physiological changes affected Lypd6 signaling, we investigated regulation of Lypd6 by environmental enrichment in mice as well as by acute or repeated exercise training (Fig. S1). Lypd6 levels were unchanged in these paradigms, suggesting at least some selectivity in the effect of nicotine on Lypd6 levels. Together with a lack of effect of knockout of nAChR subunits on Lypd6 levels, these data suggest that the effect of nicotine on Lypd6 is not dependent on nAChR levels or cholinergic tone in adult animals. It thus seems that the regulation of Lypd6 by nicotine is limited to a certain time window during early development.

Wnt/β‐catenin signaling is not only important for early development but also regulates nAChR localization and synaptic plasticity in the adult *C. elegans* nervous system (Jensen *et al*. 2012). Since both nicotine and Lypd6 protein can regulate the Wnt/β‐catenin pathway (Zhou *et al*. 2013; Özhan *et al*. 2013), it is possible that nicotine affects Lypd6 levels via the Wnt/β‐catenin pathway.

In summary, our results indicate that Lypd6 is an endogenous orthosteric antagonist of cholinergic signaling in the brain, and that it is dysregulated by nicotine exposure during early development. This suggests that part of the detrimental consequences of maternal nicotine exposure during and after gestation on development of the offspring may be because of dysregulation of Lypd6 levels.

## Supporting information


**Figure S1.** Exercise or environmental enrichment does not modify Lypd6, Lynx1, or Ly6H levels in the brain.
**Figure S2.** Characterization of Lypd6 antibody.
**Figure S3.** α7, β2, and β4 nAChRs antibodies validation.
