# Supplementary material for: Functional interaction between Lypd6 and nicotinic acetylcholine receptors
Source: J Neurochem. 2016 Aug 15;138(6):806–20. doi: 10.1111/jnc.13718 (PMC5017906; doi:10.1111/jnc.13718)

**SUPPLEMENTARY MATERIAL**

**Title:** Functional interaction between Lypd6 and nicotinic acetylcholine receptors

**Authors:** Maria Arvaniti^1^, Majbrit M. Jensen^2^, Neeraj Soni^1^, Hong Wang^3^, Anders B. Klein^1^, Nathalie Thiriet^4^, Lars H. Pinborg^2,5^, Pretal P. Muldoon^6^, Jacob Wienecke^7^, M. Imad Damaj^6^, Kristi A Kohlmeier^1^, Marjorie C. Gondré-Lewis^3^, Jens D. Mikkelsen^2^, Morten S. Thomsen^1,2, *^

**Institutional affiliation:**

1 - Department of Drug Design & Pharmacology, University of Copenhagen, Copenhagen Denmark

2 - Neurobiology Research Unit, University Hospital Copenhagen, Rigshospitalet, Copenhagen, Denmark

3 - Laboratory for Neurodevelopment, Department of Anatomy, Howard University College of Medicine. Washington D.C., USA

4 - Laboratory of Experimental and Clinical Neurosciences, University of Poitiers, Poitiers, France

5 - Epilepsy Clinic, University Hospital Copenhagen, Rigshospitalet, Copenhagen, Denmark

6 - Department of Pharmacology and Toxicology, Medical College of Virginia, Virginia Commonwealth University, Richmond, VA, USA

7 - Department of Nutrition, Exercise and Sport & Department of Neuroscience and Pharmacology, University of Copenhagen, Denmark

*** Corresponding Author:**

Morten Skøtt Thomsen, Ph.D.

Department of Drug Design and Pharmacology
Faculty of Health and Medical Sciences

University of Copenhagen
Jagtvej 160, building 22, C.421

2100 Copenhagen

Denmark
E-mail: morten.s.thomsen@sund.ku.dk

**Keywords:** nicotine, Lynx, Ly-6, LY6/PLAUR domain-containing 6, affinity purification.

## Forced exercise

Male Wistar rats (200-460 g) were acclimatized to the motorized treadmill by exposing them to a single bout of running; 30 min at 15 m/min, the day before the actual experiments started. On each training day, animals started with 5 min at 10 m/min, followed by a short break (staying at the treadmill for 2 min) and then exposed to either i) a single bout of exercise or ii) a specific training programme lasting up to 8 weeks as described in (Elfving et al., 2013). Inclination of the treadmill was 8%. Control animals were cage controls and did not perform any exercise, but were handled during weighing. Following training (acute or repeated), rats were euthanized by decapitation 24 hrs after the last training session. Frontal cortex (dissected 1.5 mm from the anterior pole, corresponding to +4.20 mm from Bregma encompassing tissue from the regions Cg1, M1, M2, and Fr3 (Paxinos and Watson) and hippocampus were dissected and kept at −80°C until further processing. The brain tissue from these animals was aliquoted and one portion has previously been analyzed (Elfving et al., 2013). The tendons of the chronic exercised rats were used in Pingel et al. (2013) which includes complete weight-scheme and training protocol (Pingel et al., 2013).

## Environmental enrichment

Male C57Bl/6J mice were housed in a temperature-controlled environment on a 12-h light/12-h dark cycle with the lights on from 7:00 a.m. to 7:00 p.m. and had ad libitum access to food and water. Animals were exposed in groups of 4 to two distinct housing environmental conditions, namely standard environments (SE) or enriched environments (EE). SE consisted of common cage housing (30x20x15 cm) while EE consisted of larger (60x38x20 cm) cages containing a running wheel, a small house and four-five toys that were changed once a week with new toys of different shapes and colors. Mice were placed in SE or EE immediately after weaning (3 weeks of age) and remained there for two months.

**Figure S1. Exercise or environmental enrichment does not modify Lypd6, Lynx1 or Ly6H levels in the brain.** The protein levels of Lypd6, Lynx1 and Ly6H was determined in the frontal cortex and hippocampus of rats subjected to forced exercise once **(A)** or repeatedly **(B)**, as well as in the frontal cortex and hippocampus of mice raised in enriched (EE) or simple environments (SE, **C**).

**Figure S2. Characterization of Lypd6 antibody.** Western blot image of recombinant human Lypd6 protein (0.001-1 μg), 38 μg rat cortex (Rat ctx) and 38 μg human cortex (Human ctx), showing that the Lypd6 antibody (#ARP53451_P050, Aviva Systems Biology) detects the recombinant Lypd6 protein at approximately 42 kDa, corresponding to the molecular weight of the recombinant GST-tagged Lypd6 (a GST tag has a molecular weight of approximately 26 kDa), and the native rat and human Lypd6 at approximately 19 kDa.

**Figure S3. α7, β2 and β4** **nAChRs antibodies validation.** Western blot images of affinity purification using human recombinant Lypd6-coupled magnetic beads on α7 **(A),** β2 **(B)** or β4 **(C)** nAChR knockout (KO) and wild type (WT) mice cortical tissue extracts. **(A)** The α7 antibody (ab23832, Abcam) detects a 56 kDa protein corresponding to the α7 nAChR subunit in both α7 WT and KO tissue in the homogenate used for affinity purification (input) and the remaining homogenate collected after pull-down (output). A band corresponding to the α7 nAChR subunit was detected in the affinity-purified sample of the α7 WT, but not in the α7 KO. **(B)** The antibody against β2 nAChR subunit (#834, Cecilia Gotti) only detects the corresponding protein at 57 kDa in homogenates and affinity-purified samples from WT cortical extracts, but not in the KO tissue. **(C)** Similarly to α7, the β4 nAChR subunit antibody (ab156213, Abcam) detects a band at 56 kDa corresponding to the β4 subunit in both β4 WT and KO homogenates (input and output), as well as in the WT affinity-purified sample, but not in the β4 KO affinity-purified sample. The α4 nAChR antibody (ab77831, Abcam) was used as a positive control in all samples, showing that α4 is co-purified with Lypd6 in all WT and KO homogenates.

## References

Elfving B, Christensen T, Ratner C, Wienecke J, Klein AB (2013) Transient activation of mTOR following forced treadmill exercise in rats. Synapse 67:620–625.

Pingel J, Wienecke J, Kongsgaard M, Behzad H, Abraham T, Langberg H, Scott A (2013) Increased mast cell numbers in a calcaneal tendon overuse model. Scand J Med Sci Sports 23:e353–e360.

##
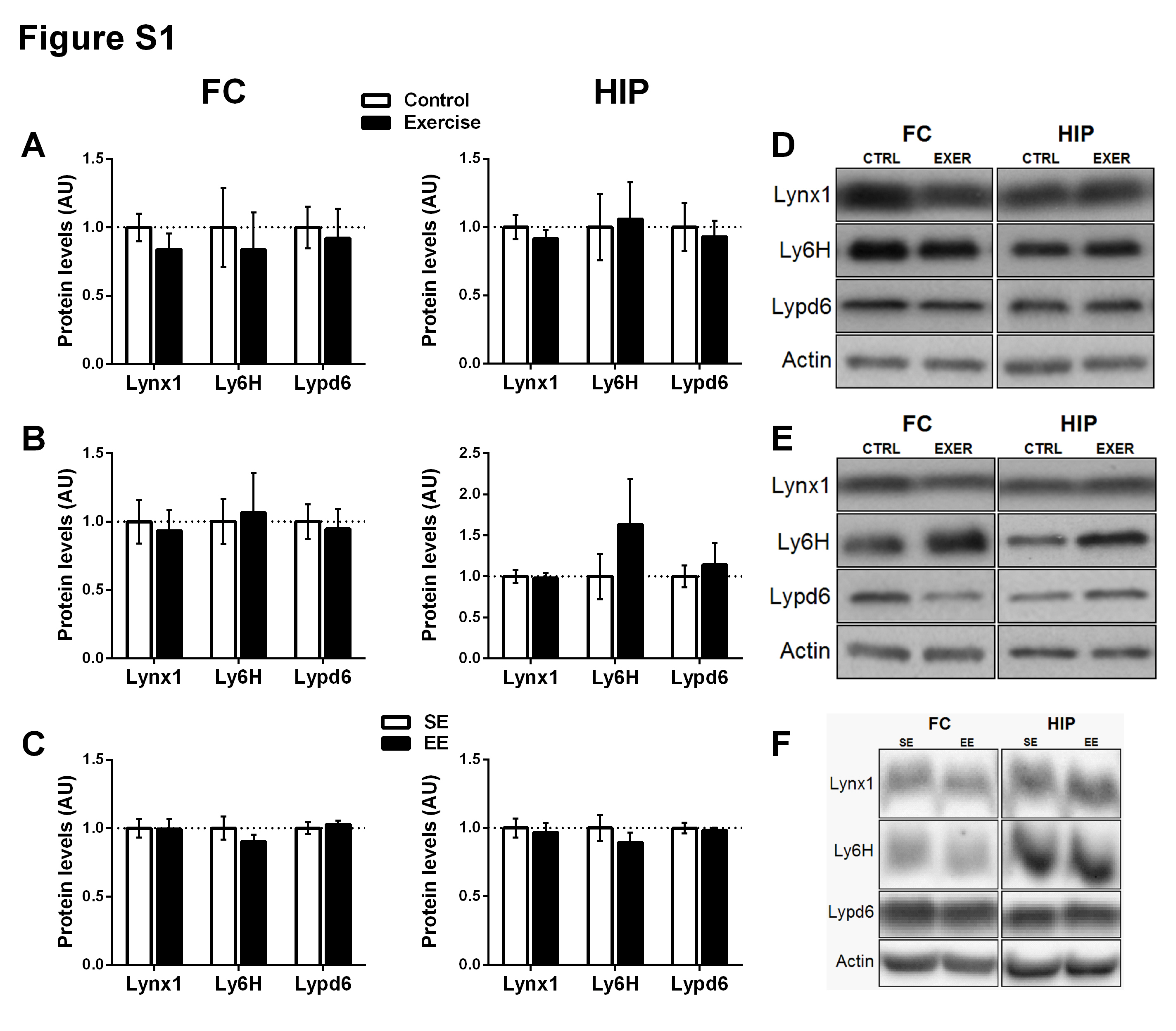


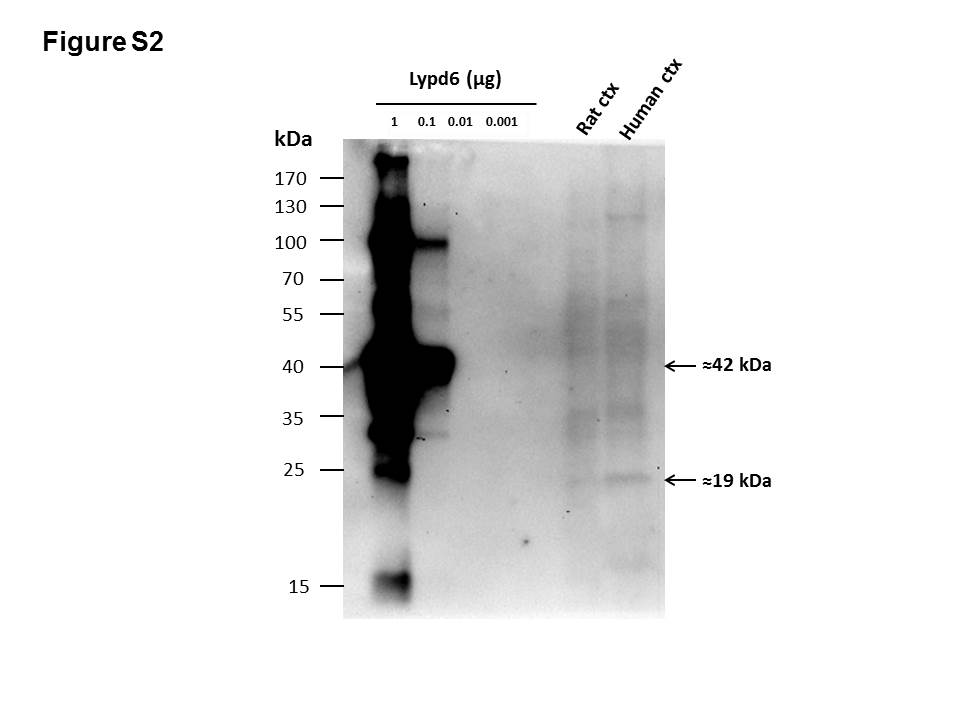


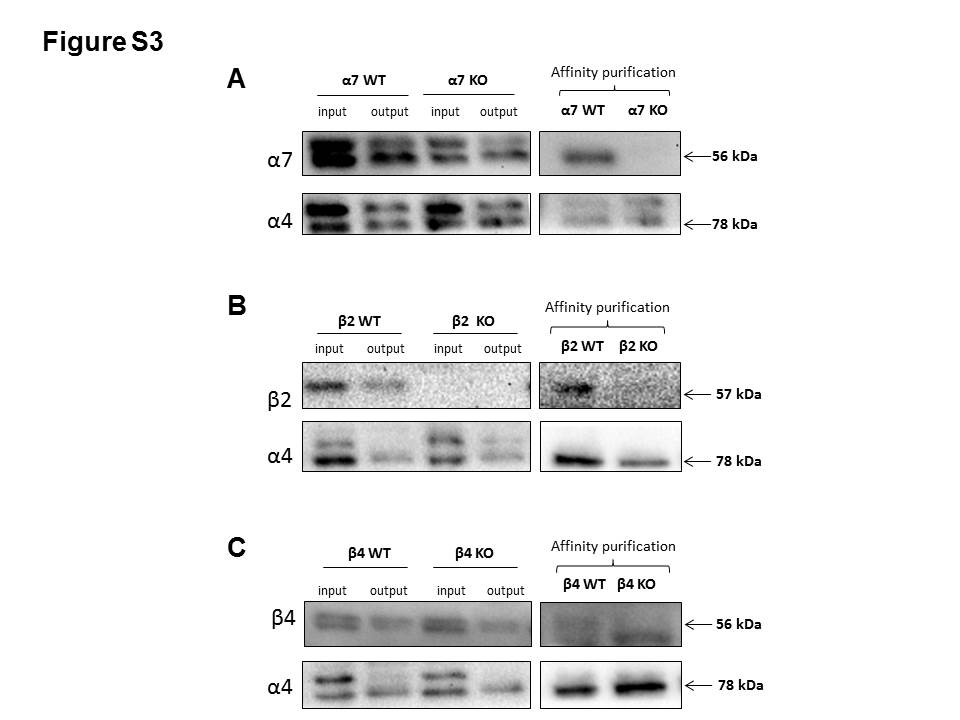

Supplement: Supplementary file 1 — Figure S1. Exercise or environmental enrichment does not modify Lypd6, Lynx1, or Ly6H levels in the brain. Figure S2. Characterization of Lypd6 antibody. Figure S3. α7, β2, and β4 nAChRs antibodies validation. [file JNC-138-806-s001.doc]
